# Supplementary material for: Return to Physical Activity in Individuals with Surgical Stomas: A Scoping Review
Source: Sports (Basel). 2024 Oct 10;12(10):273. doi: 10.3390/sports12100273 (PMC11511191; doi:10.3390/sports12100273)
Supplement: Supplementary file 1 [file sports-12-00273-s001.zip › Supplementary Table S1.pdf]

Supplementary Table S1. Search strategies.

Date Database	Search Strategies
2023 October 30 Medline	((((((((“Surgical Stomas”[Mesh] OR “Stoma, Surgical” OR “Stomata, Surgical” OR “Surgical Stomata” OR “Stomas, Surgical”) OR (“Ostomy”[Mesh] OR “Ostomies”)) OR (“Colostomy”[Mesh] OR “Colostomies”)) OR (“Ileostomy”[Mesh] OR “Ileostomies” OR “Tube Ileostomy” OR “Ileostomies, Tube” OR “Ileostomy, Tube” OR “Tube Ileostomies” OR “Incontinent Ileostomy” OR “Ileostomies, Incontinent” OR “Ileostomy, Incontinent” OR “Incontinent Ileostomies” OR “Loop Ileostomy” OR “Ileostomies, Loop” OR “Ileostomy, Loop” OR “Loop Ileostomies” OR “Continent Ileostomy” OR “Continent Ileostomies” OR “Ileostomies, Continent” OR “Ileostomy, Continent”)) OR (“Enterostomy”[Mesh] OR “Enterostomies”)) OR (“Cecostomy”[Mesh] OR “Cecostomies” OR “Tube Cecostomy” OR “Cecostomies, Tube” OR “Cecostomy, Tube” OR “Tube Cecostomies”)) OR (“Duodenostomy”[Mesh] OR “Duodenostomies”)) OR (“Jejunostomy”[Mesh] OR “Jejunostomies”)) AND (((“Sports”[Mesh] OR “Sport” OR “Athletics” OR “Athletic”) OR (“Exercise”[Mesh] OR “Exercises” OR “Physical Activity” OR “Activities, Physical” OR “Activity, Physical” OR “Physical Activities” OR “Exercise, Physical” OR “Physical Exercise” OR “Physical Exercises” OR “Acute Exercise” or “Acute Exercises” OR “Exercise, Acute” OR “Exercises, Acute” OR “Exercise, Isometric” OR “Exercises, Isometric” OR “Isometric Exercises” OR “Isometric Exercise” OR “Exercise, Aerobic” OR “Aerobic Exercise” OR “Aerobic Exercises” OR “Exercises, Aerobic” OR “Exercise Training” OR “Exercise Trainings” OR “Training, Exercise” OR “Trainings, Exercise”)) OR (“Return to Sport”[Mesh] OR “Return to Sports” OR “Sport, Return to” OR “Sports, Return to” OR “to Sport, Return” OR “to Sports, Return” OR “Return to Play” OR “Play, Return to” OR “to Play, Return” OR “Return to Sporting Activities” OR “Resumption of Sporting Activity” OR “Activity Resumption, Sporting” OR “Activity Resumptions, Sporting” OR “Sporting Activity Resumption” OR “Sporting Activity Resumptions” OR “Resumption of Recreational Activities” OR “Activities Resumption, Recreational” OR “Activities Resumptions, Recreational” OR “Recreational Activities Resumption” OR “Recreational Activities Resumptions” OR “Return to Recreational Activities”))
2023 November 9 Scopus	(INDEXTERMS (“Surgical stomas”) OR INDEXTERMS (“Colostomy”) OR INDEXTERMS (“Ileostomy”) OR INDEXTERMS (“Peritoneal Stomata”)) AND (INDEXTERMS (“Exercise”) OR INDEXTERMS (“Return to Sports”) OR INDEXTERMS (“Return to Sports”))
2023 November 13 Web of Science	TS=(“Surgical Stomas” OR “Colostomy” OR “Ileostomy” OR “Peritoneal Stomata”) AND TS=(“Return to Sports” OR “Return to Sport” OR “Exercise”)
2023 November 13 Cinahl	TX (“Surgical Stomas” OR “Colostomy” OR “Ileostomy” OR “Peritoneal stomata”) AND TX (“Return to Sport” OR “Exercise”)
2023 November 13 Lilacs	(“ESTOMA QUIRURGICO”) [Palabras] or (“ESTOMA”) or (“COLOSTOMIA”) [Palabras] and (“EJERCICIO”) or (“DEPORTE/ACTIVIDAD”) or (“EJERCICIO FISICO”) [Palabras]
2024 March 16 TripDatabase	((return AND to AND sport AND exercise AND surgical AND stomas))
2024 March 16 Epistemonikos	(title:(exercise) OR abstract:(exercise)) OR (title:("return to sport") OR abstract:("return to sport")) AND (title:("surgical stomas") OR abstract:("surgical stomas")) OR (title:(Colostomy) OR abstract:(Colostomy)) OR (title:(ileostomy) OR abstract:(ileostomy)) OR (title:("peritoneal stomata") OR abstract:("peritoneal stomata"))
