# Supplementary material for: Return to Physical Activity in Individuals with Surgical Stomas: A Scoping Review
Source: Sports (Basel). 2024 Oct 10;12(10):273. doi: 10.3390/sports12100273 (PMC11511191; doi:10.3390/sports12100273)
Supplement: Supplementary file 1 [file sports-12-00273-s001.zip › Supplementary Table S3.pdf]

Supplementary Table S3. Excluded studies

ID	Year	Journal	Title	Authors	DOI	Reason for exclusion
ID34	2023	Journal of Clinical Nursing	Ostomy-related problems and their impact on quality of life of Saudi ostomate patients: A mixed-methods study	Alenezi, Livesay, McGrath & Kimpton	10.1111/jocn.16466	No sport
ID454	2023	Pilot and feasibility studies	Hernia Active Living Trial (HALT): a feasibility study of a physical activity intervention for people with a bowel stoma who have a parastomal hernia/bulge	Munro et al	10.1186/s40814-023-01329-8	Protocol
ID81	2022	Wound Manag Prev	Health-related quality of life among patients with an ostomy regarding sex, disease diagnosis, health care provider, and ostomy type: a descriptive cross-sectional study	Alenezi, Kimpton, Livesay & McGrath	10.25270/wmp.2022.10.2027	No sport
ID628	2022	British Journal of Nursing	Gaining consensus: the challenges of living with a stoma and the impact of stoma leakage.	Aibibula et al	10.12968/bjon.2022.31.6.s30	No sport
ID382	2022	Annals of Surgery	The role of pelvic floor muscle training on low anterior resection syndrome a multicenter randomized controlled trial	Asnong et al	10.1097/SLA.0000000000005632	No sport
ID761	2022	British Journal of Community Nursing	Resuming activities with a stoma.	Burch	10.12968/bjcn.2022.27.8.392	Letter to the editor
ID2	2021	Journal of Clinical Nursing	Quality of life among ostomy patients: A narrative literature review	Alenezi, McGrath, Kimpton & Livesay	10.1111/jocn.15840	No sport
ID477	2019	Pilot and feasibility studies	A physical activity intervention to improve the quality of life of patients with a stoma: a feasibility study protocol	Hubbard et al	10.1186/s40814-019-0461-2	Protocol
ID481	2019	Annals of translational medicine	The effect of biofeedback training on intestinal function among patients with middle and low rectal cancer: a randomized controlled study	Liu et al	10.21037/atm.2019.09.62	No sport
ID443	2019	Journal of wound ostomy and continence nursing	Exercise After Ostomy Surgery and Peristomal Hernia A View from Here	Russell	10.1097/WON.0000000000000052	Opinion article
ID643	2018	British Journal of Nursing	Helping stoma patients keep active.	Fulham	10.12968/bjon.2018.27.22.S4	Letter to the editor
ID36	2017	British Journal of Nursing	Returning to the gym with a stoma: a patient's perspective	Wright		Opinion article

ID105	2016	Journal Wound Ostomy Continence Nurs	Quality of Life in US Residents with Ostomies Assessed via the SF36v2: Role-Physical, Bodily Pain, and General Health Domain	Nichols	10.1097/WON.0000000000000219	No sport
ID661	2016	Gastrointestinal Nursing	Inspiring confidence: helping a young ostomate return to sport.	Sica	10.12968/gasn.2016.14.4.16	Opinion article
ID604	2014	Journal of Community Nursing	Ensuring optimum quality of life in community patients with a stoma.	Burch	http://www.jcn.co.uk/journal/01-2014/	No sport
ID190	2011	Colorectal Dis	Function after intersphincteric resection for low rectal cancer and its influence on quality of life	Barisic et al	10.1111/j.1463-1318.2010.02244.x	No sport
ID455	2009	British journal of nursing	A healthy lifestyle for the ostomist in relation to exercise.	Varma	10.12968/bjon.2009.18.Sup6.44171	Opinion article
ID371	2008	Current Opinion in Critical Care	From rehabilitation to optimal function: Role of clinical exercise therapy	Storch & Kruszynski	10.1097/MCC.0b013e328306ef13	No surgical stoma
ID546	2005	Supportive Care in Cancer	Barriers and facilitators of exercise experienced by cancer survivors: a mixed methods systematic review.	Clifford et al	10.1007/s00520-017-3964-5	No surgical stoma
ID630	2005	Oncology Nursing Forum	Gender Differences in Quality of Life Among Long-Term Colorectal Cancer Survivors with Ostomies.	Grant et al	10.1188/11.ONF.587-596	No sport
ID13	2004	Ostomy Wound Manage	Exercise and ostomy	Turnbull	PMID: 15206087	Grey Literature
ID293	1989	ANNA Journal	Use of an ostomy pouch for pediatric CAPD swimmers	Sandahl & Owens	PMID: 2742395	Opinion article
