# Supplementary material for: Return to Physical Activity in Individuals with Surgical Stomas: A Scoping Review
Source: Sports (Basel). 2024 Oct 10;12(10):273. doi: 10.3390/sports12100273 (PMC11511191; doi:10.3390/sports12100273)
Supplement: Supplementary file 1 [file sports-12-00273-s001.zip › Supplementaty Table S2.pdf]

**Supplementary Table S2.** Critical appraisal of the included studies

| Author (year); Design | Q1 | Q2 | Q3 | Q4 | Q5 | Q6 | Q7 | Q8 | Q9 | Q10 | Q11 | Q12 | Q13 | N (%) |
|---|---|---|---|---|---|---|---|---|---|---|---|---|---|---|
| Park et al (2023); Cross-sectional [26] | D | Y | Y | Y | N | N | Y | Y | - | - | - | - | - | 5/8 (62.5) |
| Goodman et al (2022); Cross-sectional [27] | Y | D | Y | Y | N | N | Y | Y | - | - | - | - | - | 5/8 (62.5) |
| Krogsgaard et al (2022); Cross-sectional [28] | Y | Y | Y | Y | Y | Y | Y | Y | - | - | - | - | - | 8/8 (100) |
| Mo et al (2021); Cross-sectional [29] | Y | Y | Y | Y | Y | Y | Y | Y | - | - | - | - | - | 8/8 (100) |
| Saunders & Brunet (2019); Qualitative [30] | Y | Y | Y | Y | N | N | Y | Y | Y | - | - | - | - | 7/9 (77.7) |
| Lowe, Alsaleh & Blake (2019); Cross-sectional [31] | Y | Y | Y | Y | Y | Y | Y | Y | - | - | - | - | - | 8/8 (100) |
| Kindred et al (2019); RCT [32] | Y | Y | Y | Y | Y | D | Y | Y | D | Y | Y | Y | Y | 11/13 (84.6) |
| Russell (2017); Cross-sectional [33] | N | Y | D | D | NA | NA | D | Y | - | - | - | - | - | 2/8 (25) |
| Russell (2017); Cross-sectional [34] | N | Y | D | D | NA | NA | D | Y | - | - | - | - | - | 2/8 (25) |
| Krouse et al (2017); Cross-sectional [35] | Y | Y | Y | Y | Y | Y | Y | Y | - | - | - | - | - | 8/8 (100) |
| Wiskemann et al. (2016); Case report [36] | Y | Y | Y | Y | Y | Y | Y | Y | - | - | - | - | - | 8/8 (100) |
| Sica (2016); Case report [37] | D | Y | Y | D | N | Y | Y | Y | - | - | - | - | - | 5/8 (62.5) |
| Anderson et al (2013); Qualitative [38] | Y | Y | D | Y | Y | D | Y | Y | Y | - | - | - | - | 7/9 (77.7) |
| Courneya et al (1999); Cohort [39] | Y | Y | Y | Y | Y | D | D | D | D | D | D | - | - | 5/11 (45.4) |
| Isaacs (1984); Case series [40] | Y | D | Y | D | N | Y | Y | Y | Y | N | - | - | - | 6/10 (60) |

*Qualitative* (Q1: Is there congruity between the stated philosophical perspective and the research methodology?; Q2: Is there congruity between the research methodology and the research question or objectives?; Q3: Is there congruity between the research methodology and the methods used to collect data?; Q4: Is there congruity between the research methodology and the representation and analysis of data?; Q5: Is there congruity between the research methodology and the interpretation of results?; Q6: Is there a statement locating the researcher culturally or theoretically?; Q7: Is the influence of the researcher on the research, and vice- versa, addressed?; Q8: Are participants, and their voices, adequately represented?; Q9: Is the research ethical according to current criteria or, for recent studies, and is there evidence of ethical approval by an appropriate body?; Q10: Do the conclusions drawn in the research report flow from the analysis, or interpretation, of the data?).

*Cross-sectional* (Q1: Were the criteria for inclusion in the sample clearly defined?; Q2: Were the study subjects and the setting described in detail?; Q3: Was the exposure measured in a valid and reliable way?; Q4: Were objective, standard criteria used for measurement of the condition?; Q5: Were confounding factors identified?; Q6: Were strategies to deal with confounding factors stated?; Q7: Were the outcomes measured in a valid and reliable way?; Q8: Was appropriate statistical analysis used?).

*RCT* (Q1: Was true randomization used for assignment of participants to treatment groups?; Q2: Was allocation to treatment groups concealed?; Q3: Were treatment groups similar at the baseline?; Q4: Were participants blind to treatment assignment?; Q5: Were those delivering treatment blind to treatment assignment?; Q6: Were outcomes assessors blind to treatment assignment?; Q7: Were treatment groups treated identically other than the intervention of interest?; Q8: Was follow up complete and if not, were differences between groups in terms of their follow up adequately described and analyzed?; Q9: Were participants analyzed in the groups to which they were randomized?; Q10: Were outcomes measured in the same way for treatment groups?; Q11: Were outcomes measured in a reliable way?; Q12: Was appropriate statistical analysis used?; Q13: Was the trial design appropriate, and any deviations from the standard RCT design (individual randomization, parallel groups) accounted for in the conduct and analysis of the trial?).

*Cohort* (Q1: Were the two groups similar and from the same population; Q2: Were exposures measured in a similar way to allocate people to the exposed and unexposed groups; Q3: Was exposure measured in a valid and reliable way; Q4: Were confounders identified; Q5: Were strategies for dealing with confounders indicated; Q6: Were the groups/participants free of the outcome at baseline (or at the time of exposure); Q7: Was follow-up time reported and was it sufficient to produce results? Q7: Was follow-up time reported and was it sufficient to produce results; Q8: Was follow-up time reported and was it sufficient to produce results; Q9: Was follow-up completed and, if not, were the reasons for loss to follow-up described and discussed; Q10: Were strategies used to address incomplete follow-up; Q11: Was appropriate statistical analysis used)?
*Case report* (Q1: Were the patient's demographic characteristics clearly described; Q2: Was the patient's history clearly described and presented in the form of a chronology; Q3: Was the patient's current clinical status at the time of presentation clearly described; Q4: Were the diagnostic tests or assessment methods and results clearly described; Q5: Were the intervention(s) or treatment procedure(s) clearly described; Q6: Were the post-intervention clinical status clearly described; Q7: Were the intervention(s) or treatment procedure(s) clearly described? Q5: Was the intervention(s) or treatment procedure(s) clearly described; Q6: Was the post-intervention clinical status clearly described; Q7: Were adverse events (harms) or unanticipated events identified and described; Q8: Does the report provide lessons for implementation)?
*Case series* (Q1: Were there clear criteria for inclusion in the case series; Q2: Was the condition measured in a standard and reliable way in all participants included in the case series; Q3: Were valid methods for disease identification used in all participants included in the case series; Q4: Did the case series have consecutive inclusion of participants; Q5: Were all participants included in the case series; Q6: Were demographics of participants in the study clearly reported; Q7: Was clinical information of participants clearly reported; Q8: Were results or follow-up of cases clearly reported; Q9: Were results or follow-up of cases clearly reported? Q6: Were the demographics of the study participants clearly reported; Q7: Was the clinical information of the participants clearly reported; Q8: Were the outcomes or follow-up of the cases clearly reported; Q9: Were the demographics of the submitting site(s) clearly reported; Q10: Was the statistical analysis adequate)?
Y: Yes; N: No; D: Doubt, NA: Not Applicable
